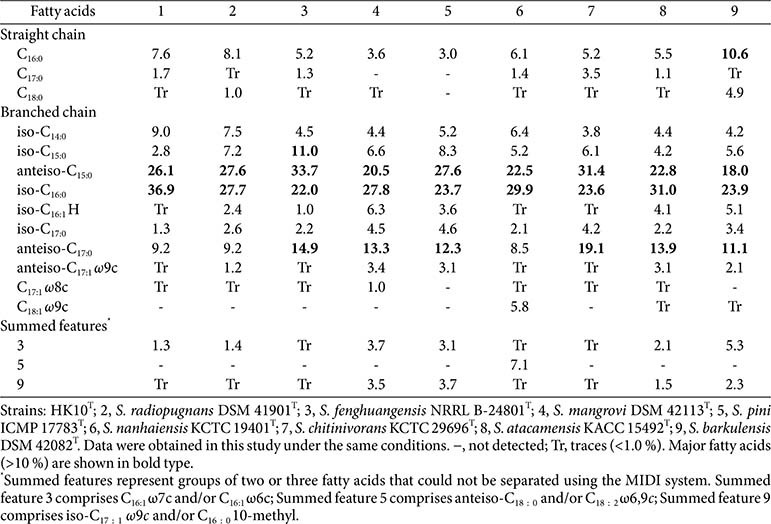# *Streptomyces shinuiensis* sp. nov., a Salternamides-Producing Bacterium Isolated from Saltern Sediment

**DOI:** 10.4014/jmb.2510.10026

**Published:** 2025-12-18

**Authors:** Yeonjung Lim, Ye-Eun Moon, Mirae Kim, Jayho Lee, Dong-Chan Oh, Jang-Cheon Cho

**Affiliations:** 1 Department of Biological Sciences and Bioengineering, Inha University, Incheon 22212, Republic of Korea; 2 College of Pharmacy, Seoul National University, Seoul 08826, Republic of Korea; 3 Natural Products Research Institute and Research Institute of Pharmaceutical Sciences, College of Pharmacy, Seoul National University, Seoul 08826, Republic of Korea

**Keywords:** *Streptomyces shinuiensis*, novel species, saltern sediment, marine actinobacterium, genome, polyphasic taxonomy

## Abstract

Strain HK10^T^, previously isolated from saltern sediment and identified as a salternamides-producing bacterium, was taxonomically characterized in the present study. The strain is a Gram-stain-positive, aerobic, and filamentous actinobacterium. Phylogenetic analysis based on 16S rRNA gene sequences revealed that strain HK10^T^ belongs to the genus *Streptomyces* and is most closely related to *Streptomyces radiopugnans* R97^T^ (99.4% sequence similarity), followed by other *Streptomyces* species with sequence similarities of ≤98.9%. The draft genome sequence of strain HK10^T^ was 6.69 Mbp and a DNA G+C content of 72.7%. Average nucleotide identity (ANI) and digital DNA–DNA hybridization (dDDH) values between strain HK10^T^ and type strains of the genus *Streptomyces* were ≤92.7% and ≤53.0%, respectively, which are below the thresholds for species delineation, indicating that strain HK10^T^ represents a novel species. The major cellular fatty acids were iso-C_16:0_, anteiso-C_15:0_, anteiso-C_17:0_, and iso-C_14:0_. The predominant menaquinone was menaquinone-9 (MK-9). The polar lipid profile included phosphatidylethanolamine, phosphatidylglycerol, and phosphatidylinositol dimannoside. Whole-cell hydrolysates of strain HK10^T^ contained *_L,L_*-diaminopimelic acid and glycine. Based on phylogenetic, genomic, chemotaxonomic, and physiological evidence, strain HK10^T^ is considered to represent a novel species of the genus *Streptomyces*, for which the name *Streptomyces shinuiensis* sp. nov. is proposed. The type of strain is HK10^T^ (= NBRC 114904^T^ = KACC 22137^T^).

## Introduction

The genus *Streptomyces*, a member of the family *Streptomycetaceae*, comprises the largest number of validly published named species in the phylum *Actinomycetota*. First described by Waksman and Henrici [1], *Streptomyces* species are found in diverse habitats, exhibiting remarkable versatility and adaptability to varying environmental conditions [2]. As of April 2025, the genus comprises at least 743 species with validly published and correct names, according to the List of Prokaryotic names with Standing in Nomenclature (LPSN) [3]. Members of the genus *Streptomyces* are typically aerobic Gram-positive bacteria with high genomic G+C content that are renowned for producing a broad range of secondary metabolites, including antibiotics and hydrolytic enzymes [4, 5]. Notably, *Streptomyces* species are responsible for synthesizing over two-thirds of clinically useful antibiotics of natural origin, such as streptomycin and neomycin [6]. This extensive biosynthetic capacity has established *Streptomyces* as a cornerstone in pharmaceutical development and biotechnology [7].

In this study, we focus on *Streptomyces* sp. HK10^T^, a halophilic bacterium isolated from a high-salinity saltern environment. Strain HK10^T^ has been identified as a producer of salternamides A–E, representing the first discovery of bioactive compounds from saltern-derived actinomycetes [8-10]. Subsequent studies have demonstrated that these compounds exhibit potent *in vitro* cytotoxicity against cancer cells by inhibiting the hypoxia-induced accumulation of HIF-1α, leading to G2/M cell cycle arrest and apoptosis [9]. Here, we present a comprehensive taxonomic characterization of strain HK10^T^, employing phylogenetic, physiological, and chemotaxonomic analyses to establish its status as a novel species of the genus *Streptomyces*, isolated from saltern sediment.

## Materials and Methods

### Isolation and Strains

Strain HK10^T^ was originally isolated from saltern sediment on Shinui Island, Republic of Korea (34°36'48.0"N, 126°04'32.5"E) using actinomycete isolation agar [8]. Subsequent studies further demonstrated that this actinobacterium produces salternamides [8, 10]. For taxonomic characterization, the strain was revived from frozen glycerol stocks and routinely cultured on International *Streptomyces* Project medium 2 (ISP2; BD Diagnostics, USA), prepared with 750 mL of aged seawater (2.5% salinity) and 250 mL of distilled water. Aged seawater (2.5% salinity) was prepared by storing natural seawater at room temperature for more than one month prior to use, in accordance with standard laboratory practice.

To compare the phenotypic characteristics of strain HK10^T^ with those of its closest phylogenetic neighbors, eight type strains were obtained from various culture collections. These included *Streptomyces radiopugnans* DSM 41901^T^, *S. barkulensis* DSM 42082^T^ and *S. mangrovi* DSM 42113^T^ from the Leibniz Institute-German Collection of Microorganisms (DSMZ); *S. fenghuangensis* NRRL B-24801^T^ from the ARS Culture Collection (NRRL); *S. pini* ICMP 17783^T^ from the International Collection of Microorganisms from Plants (ICMP); *S. nanhaiensis* KCTC 19401^T^ and *S. chitinivorans* KCTC 29696^T^ from the Korean Collection for Type Cultures (KCTC); and *S. atacamensis* KACC 15492^T^ from the Korean Agricultural Culture Collection (KACC). Each strain was revived according to the respective culture collection’s protocol and subsequently maintained on ISP2 medium at 30°C for 5 days, under the same conditions as strain HK10^T^.

### 16S rRNA Gene Sequencing and Phylogenetic Analysis

Genomic DNA of strain HK10^T^ was extracted using the DNeasy Blood & Tissue Kit (Qiagen, Germany). The 16S rRNA gene was amplified by PCR using universal bacterial primers 27F and 1492R and sequenced via Sanger sequencing (Macrogen Inc., Republic of Korea) using the sequencing primers 27F, 518F, 800R, and 1492R. The resulting 16S rRNA gene sequence of strain HK10^T^ (GenBank accession number PV018052) was analyzed using a BLASTn search against the GenBank database and through pairwise sequence comparisons in the EzBioCloud database [11]. For phylogenetic analysis, the 16S rRNA gene sequence of strain HK10^T^, along with those of closely related taxa, was aligned using the SILVA Incremental Aligner (SINA) [12]. Phylogenetic trees were constructed from the aligned sequences using MEGA 12 [13] with three algorithms: the maximum-likelihood method based on the Tamura–Nei model, the neighbor-joining method with the Jukes–Cantor correction, and the minimum-evolution method, also using the Jukes–Cantor correction. The robustness of the resulting trees was evaluated by bootstrap analysis based on 1,000 replicates.

### Genome Sequencing, Assembly, Annotation, and Analysis

Whole-genome sequencing of strain HK10^T^ was performed using a hybrid approach combining long-read and short-read sequencing technologies. A 20 kb SMRTbell library was prepared and sequenced using the PacBio RS II platform (Pacific Biosciences, USA), and short reads were generated using the Illumina NovaSeq 6000 platform (2 × 150 bp paired-end; DNA Link Inc., Republic of Korea) with a TruSeq Nano DNA library preparation kit. Raw Illumina reads were trimmed using BBDuk with the following parameters: ktrim=r, k=23, mink=11, hdist=1, tpe, tbo, ftm=5, qtrim=rl, trimq=10, and minlen=80. Hybrid genome assembly was carried out using Hybracter (v0.7.3) [14] with the options --no_medaka --flyeModel --pacbio-raw. Genome coverage was calculated by mapping PacBio long reads to the assembled genome using SAMtools (v1.18) [15]. CheckM (v1.1.3) was employed to evaluate the completeness and contamination levels of the assembled genome [16].

To facilitate comparative genomic analyses and assess genomic relatedness, whole-genome sequencing of *S. mangrovi* DSM 42113^T^ was performed using the Illumina NovaSeq 6000 platform (2 × 150 bp; Macrogen Inc., Republic of Korea). In addition, the genomes of *S. atacamensis* KACC 15492^T^, *S. chitinivorans* KCTC 29696^T^, and *S. nanhaiensis* KCTC 19401^T^ were sequenced using the Illumina MiSeq platform (2 × 300 bp; ChunLab Inc., Republic of Korea). The genome of *S. fenghuangensis* NRRL B-24801^T^ was sequenced using the Illumina HiSeq X Ten platform (2 × 150 bp; Macrogen Inc.). For the strains sequenced in this study, assembly statistics including N50, L50, and the largest contig size were calculated using QUAST (v 5.3.0) [17] to assess the quality of the hybrid assembly (Table S1). For genome-based analyses (Fig. 2, Tables 1 and S3), the publicly available genome sequences of the type strains *S. radiopugnans* CGMCC 4.3519^T^ (FOET00000000), *S. pini* PL19^T^ (FOSG01000050), and *S. barkulensis* RC 1831^T^ (PGSG00000000) were retrieved from the GenBank database. Average nucleotide identity (ANI) values were calculated using the Enveomics toolkit [18] and digital DNA–DNA hybridization (dDDH) values were estimated using the Genome-to-Genome Distance Calculator (GGDC 2.1) [19].

To infer a genome-based phylogenetic tree, 81 universal bacterial core genes were extracted using the up-to-date bacterial core gene set and pipeline (UBCG2) [20] and were used to reconstruct a phylogenomic tree using RAxML (v8.2.12) [21] with a PROTGAMMAAUTO model including 100 bootstrap iterations. For the reconstruction of metabolic pathways, each genome was annotated with Prokka (v1.14.6) [22] and the resultant protein sequences were queried in BlastKOALA based on KEGG pathway database [23] followed by KofamKOALA [24]. For the analysis of the distribution of Clusters of Orthologous Groups (COG) categories [25], the protein sequences were searched against the COG database using RPS-BLAST (e-value cutoff: 0.01) [26]. Biosynthetic gene clusters (BGCs) for secondary metabolite were screened using antiSMASH (v7.1.0) [27].

### Physiological and Chemotaxonomic Characterization

Cultural features were assessed on various media, including the International *Streptomyces* Project (ISP) series media [28], Czapek’s agar (CZA), Gause’s synthetic agar no. 1, and marine agar 2216 (MA). For scanning electron microscopy, cells of strain HK10^T^ grown in ISP2 broth and on ISP2 agar were harvested after incubation at 25°C for the appropriate periods (5 days for broth cultures and 20 days for agar cultures). Samples were processed and examined using a scanning electron microscope (S-4300SE, Hitachi, Japan). Gram-staining characteristics were examined using the Ryu non-staining KOH method [29]. The temperature range and optimum for growth were determined in marine ISP2 medium incubated at 4°C, 10–30°C (in 5°C intervals), 37°C, 41–45°C (in 1°C intervals), and 50°C. Tolerance to salinity was evaluated by supplementing NaCl-free marine ISP2 with 0–20% of NaCl in 1% increments. The pH range and optimum were assessed in marine ISP2 adjusted to pH 4.0–12.0 (in 1.0 pH unit intervals). Hydrolytic activity against various macromolecules was evaluated on marine ISP2 agar supplemented with starch (1%, w/v), CM-cellulose (1%, w/v), casein (3% skim milk, w/v), colloidal chitin (1%, w/v), elastin (0.2%, w/v), Tween 20 (1%, v/v), and Tween 80 (1%, v/v). DNase activity was tested using DNase test agar (BD Diagnostics, USA). Hydrogen sulfide production was assessed using Triple Sugar Iron Agar (BD Diagnostics). Additional biochemical characteristics were determined using API 20NE and API ZYM strips (bioMérieux, France), and the Biolog GEN III MicroPlate system (Biolog), following the manufacturer’s protocols with the salinity adjusted to 2%.

For fatty acid methyl ester (FAME) analysis, biomass of HK10^T^ and closely related type strains of the genus *Streptomyces* was harvested from colonies grown on marine ISP2 at 30°C for 5 days. FAME profiles were analyzed by gas chromatography (Agilent 7890 GC system, USA) using the Sherlock Microbial Identification System version 6.1 (MIDI) with the TSBA6 database [30]. Polar lipids were extracted following the method described by Minnikin *et al*. [31] and separated by two-dimensional thin-layer chromatography (TLC) on silica gel 60 F_254_ plates. All polar lipids were visualized using 10% (w/v) phosphomolybdic acid spray. Specific lipid classes were identified by applying selective detection reagents: 0.2% (w/v) ninhydrin for aminolipids, 1.3% (w/v) molybdenum blue for phospholipids, 2.4% (w/v) α-naphthol for glycolipids, and Dragendorff reagent for phosphatidylcholine. Menaquinones were extracted according to the protocol of Collins *et al*. [32] and analyzed using reverse-phase partition chromatography on Merck HPTLC RP-18 F_254_ plates [33]. Amino acids were extracted from whole-cell hydrolysates, purified, and separated on TLC cellulose F_25_ aluminum sheets, as previously described [34]. Amino acids were detected with 0.2% (w/v) ninhydrin in water-saturated butanol.

## Results and Discussion

### Phylogenetic Analysis Based on 16S rRNA Gene Sequences

**Fig. 1:**
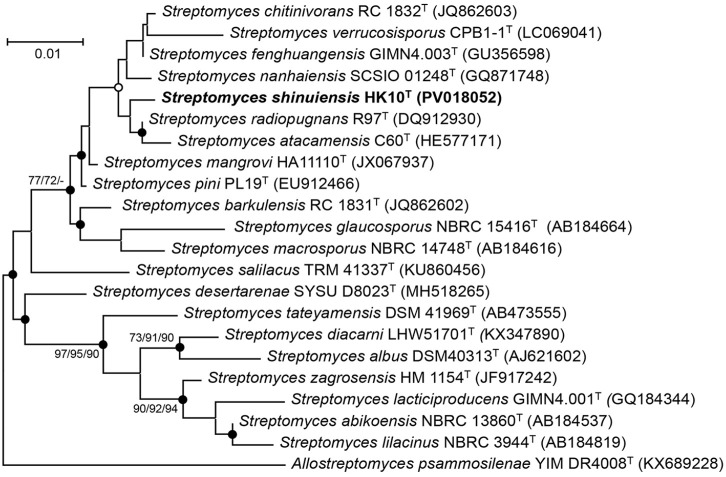
Maximum-likelihood phylogenetic tree based on 16S rRNA gene sequences showing the relationships between strain HK10^T^ and related type strains of the genus *Streptomyces*. Bootstrap values (expressed as percentages of 1000 replications) are shown at nodes for maximum-likelihood, neighbor-joining, and minimum-evolution methods, respectively. *Allostreptomyces psammosilenae* YIM DR4008^T^ (KX689228) was used as an outgroup. Filled circles indicate that the corresponding nodes were recovered by all treeing methods. Open circles indicate that the corresponding nodes were recovered by any two out of three. Bar, 0.01 substitutions per nucleotide position.

Sequence similarity analyses based on the 16S rRNA gene sequence indicated that strain HK10^T^ belongs to the genus *Streptomyces*. The strain exhibited the highest sequence similarity to *S. radiopugnans* R97^T^ (99.4%), followed by *S. fenghuangensis* GIMN4.003^T^ (98.9%), *S. mangrovi* HA11110^T^ (98.8%), *S. pini* PL19^T^ (98.8%), *S. nanhaiensis* SCSIO 01248^T^ (98.8%), *S. chitinivorans* RC 1832^T^ (98.7%), *S. atacamensis* C60^T^ (98.7%), and *S. barkulensis* RC 1831^T^ (98.2%) (Table 1). Phylogenetic analysis using three tree-building algorithms consistently placed strain HK10^T^ in a distinct clade with these closely related type strains within the genus *Streptomyces* (Fig. 1), supporting its taxonomic affiliation with this genus. However, as the 16S rRNA gene sequence similarity between strain HK10^T^ and several type strains exceeded 98.7% threshold, whole-genome analysis was required to confirm its taxonomic status as a novel species.

### Phylogenomic Analysis and Genomic Characteristics

The draft whole-genome sequence of strain HK10^T^ consisted of eight contigs, with a total length of 6,688,022 bp, a genome coverage of 204.5×, and a DNA G+C content of 72.7% (Table 1). Genome completeness and contamination were estimated at 97.7% and 1.3%, respectively, using CheckM. The annotated genome contained six copies of the 16S rRNA gene, all of which were identical to the PCR-amplified sequence. Genome relatedness analyses revealed ANI and dDDH values of 92.2% and 49.9%, respectively, between strain HK10^T^ and *S. radiopugnans* DSM 41901^T^ (Table 1). The corresponding values between HK10^T^ and other related *Streptomyces* species ranged from 92.0–92.7% (ANI) and 49.1–53.0% (dDDH), all well below the established species delineation thresholds of 95–96% for ANI and 70% for dDDH [35], and particularly below the recently proposed 96.7% ANI threshold for *Streptomyces* [36].

**Fig. 2:**
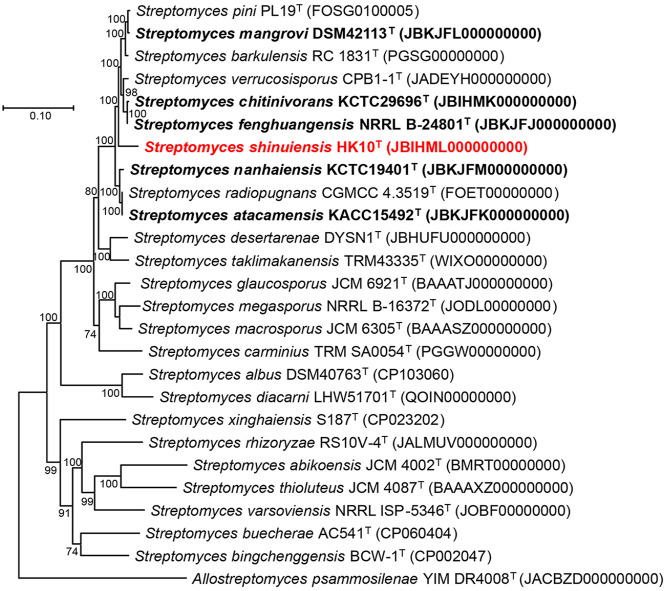
Phylogenomic tree of strain HK10^T^ and related species of the genus *Streptomyces*. The tree was constructed using RAxML (v8.2.12) with the PROTGAMMAAUTO model, based on a concatenated alignment of core genes extracted using the UBCG2 pipeline. The genomes shown in bold were sequenced in the present study. *Allostreptomyces psammosilenae* YIM DR4008^T^ was used as an outgroup. Bootstrap supporting values (100 iterations) are indicated on the nodes. Bar, 0.10 substitution per amino acid position.

These results support the conclusion that strain HK10^T^ represents a novel species within the genus *Streptomyces*. In the phylogenomic tree, strain HK10^T^ was positioned within the clade containing closely related species, including *S. radiopugnans* DSM 41901^T^ (the strain with the highest 16S rRNA similarity) and several other *Streptomyces* species whose genome were sequenced in this study (Fig. 2). This phylogenomic inference further confirmed the affiliation of strain HK10^T^ within the genus *Streptomyces*.

The general genomic features of strain HK10^T^ and other *Streptomyces* genomes are summarized in Table 1. The genome of strain HK10^T^ encodes a variety of carbon metabolic pathways, including the Embden-Meyerhof-Parnas (EMP) pathway, gluconeogenesis, the pentose phosphate pathway (PPP), the tricarboxylic acid (TCA) cycle, and 5-phospho-α-D-ribose-1-diphosphate (PRPP) biosynthesis, all of which are commonly found in *Streptomyces* species. In addition, genes involved in nitrogen and sulfur-related metabolism, such as dissimilatory nitrate reduction and assimilatory sulfate reduction, were also identified.

The secondary metabolite biosynthetic potential of strain HK10^T^, as determined by antiSMASH v7.1.0, comprised 26 predicted biosynthetic gene cluster (BGC) regions corresponding to 46 BGC types (Table S2). Among these, six BGCs exhibited >50% similarity to MiBIG reference clusters, including a type II polyketide synthase (PKS) (region 1.4), an unclassified ribosomally synthesized and post-translationally modified peptide (RiPP) (region 1.7), a nonribosomal peptide synthetase (NRPS)-like fragment (region 1.10), lipolanthine (region 1.16), ectoine (region 1.17), and an NRPS-independent (NI) siderophore cluster (region 1.20) (Table S2). These clusters showed 66–100% similarity to MiBIG reference entries involved in the biosynthesis of spore pigment, streptamidine, sporolide A or B, SapB, ectoine, and legonoxamine A/desferrioxamine B/legonoxamine B. Notably, a highly reducing type II PKS (HR-T2PKS) cluster (region 4.1), putatively responsible for the biosynthesis of salternamides A–D, which are chlorinated compounds in the manumycin family with potent *in vitro* cytotoxicity against cancer cells [8], was identified in the genome (Table S2). This HR-T2PKS cluster, spanning 41.9 kb, showed similarity to the BGC of asukamycin [37], another compound in the manumycin family, and contained genes encoding 3-amino-4-hydroxybenzoic acid synthase, flavin-dependent monooxygenases, flavin reductases, hydratases, dehydrogenases, isomerases, and other tailoring enzymes. These findings highlight the strain’s genetic capacity for diverse secondary metabolite biosynthesis. Nevertheless, the predicted BGCs represent genomic potential, and actual metabolite production must be validated experimentally.

The identification of orthologous genes based on the COG database revealed that the majority of genes (≥5.0%) in the genome of strain HK10^T^ were assigned to functional categories including transcription (9.1%), carbohydrate transport and metabolism (7.8%), general function prediction only (7.8%), signal transduction mechanisms (7.5%), amino acid transport and metabolism (7.4%), translation, ribosomal structure, and biogenesis (6.6%), coenzyme transport and metabolism (6.5%), lipid transport and metabolism (6.2%), and energy production and conversion (6.1%). These distributions were comparable to those observed in the closely related strain *S. radiopugnans* CGMCC 4.3519^T^ (Table S3).

### Physiology and Chemotaxonomic Characteristics

**Fig. 3:**
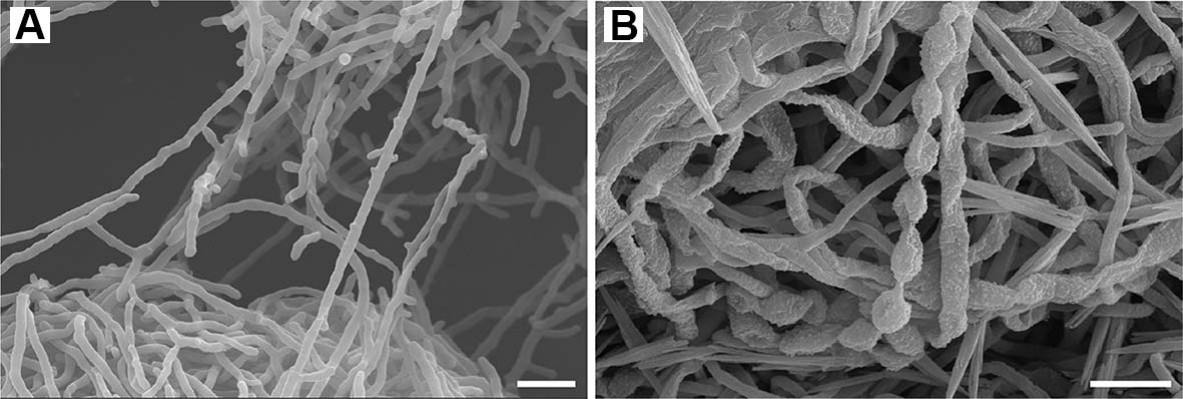
Scanning electron micrographs of strain HK10^T^. (**A**) Branched substrate mycelia with straight to flexuous hyphae observed after cultivation in marine ISP2 broth for 5 days at 25°C. (**B**) Aerial hyphae grown on marine ISP2 agar for 20 days at 25°C, differentiated into oval-shaped spores arranged in straight to curved chains with a warty surface ornamentation. Scale bars = 2.5 μm.

Cells of strain HK10^T^ were Gram-stain-positive, aerobic, non-motile, catalase-negative, and oxidase-positive. Scanning electron micrographs of ISP2 broth-grown cultures for five days revealed well-developed, branched substrate mycelia composed of straight to flexuous hyphae (Fig. 3A). On ISP2 agar, the strain produced yellow-colored substrate mycelia and white to grey aerial mycelia. After incubation for 20 days at 25°C, the aerial hyphae differentiated into oval-shaped spores (approximately 1.0 × 1.5 μm) arranged in straight to curved chains, appearing to comprise approximately 10–30 spores, with a warty surface ornamentation (Fig. 3B). The morphological, cultural, physiological, and biochemical characteristics of strain HK10^T^ are presented in Tables 2, S4, S5, and the species protologue. Strain HK10^T^ exhibited phenotypic differences compared to other closely related *Streptomyces* species, including spore chain morphology, growth range, various enzyme activities, macromolecule degradation (Table 2), and carbon source oxidation patterns (Table S4). For example, strain HK10^T^ oxidized D-cellobiose, sucrose, D-raffinose, α-D-glucose, L-rhamnose, D-sorbitol, gelatin, L-lactic acid, and α-hydroxybutyric acid, distinguishing it from the related type strains.

The major cellular fatty acids (>10%) were iso-C_16:0_ (36.9%) and anteiso-C_15:0_ (26.0%) (Table 3), consistent with those found in other species of the genus *Streptomyces*. The major polar lipids included phosphatidylethanolamine (PE), diphosphatidylglycerol (DPG), phosphatidylinositol dimannoside (PIDM), along with two unknown phospholipids, an unknown glycolipid, and two unidentified lipids (Fig. S1), displaying a polar lipid profile generally similar to those of closely related *Streptomyces* species. The predominant respiratory quinone detected in strain HK10^T^ was menaquinone-9 (MK-9), in agreement with its closely related *Streptomyces* species. The cell wall amino acid composition of strain HK10^T^ contained *_L,L_*-diaminopimelic acid and glycine, characteristic of cell wall chemotype I, which is commonly observed in the genus *Streptomyces*. Collectively, these chemotaxonomic features support the classification of strain HK10^T^ within the genus *Streptomyces*.

### Taxonomic Conclusion

Phylogenetic and phylogenomic analyses, together with genomic and chemotaxonomic data, support the assignment of strain HK10^T^ to the genus *Streptomyces*. Although strain HK10^T^ shares a high 16S rRNA gene sequence similarity (>98.7%) with several type strains of the genus *Streptomyces*, including 99.4% with *S. radiopugnans* DSM 41901^T^, the ANI and dDDH values between HK10^T^ and its closest relatives fall below the accepted thresholds for prokaryotic species delineation, indicating that the strain represents a distinct species. In addition, strain HK10^T^ can be differentiated from related *Streptomyces* species based on a combination of physiological and biochemical characteristics. On the basis of these results, strain HK10^T^ is considered to represent a novel species of the genus *Streptomyces*, for which the name *Streptomyces shinuiensis* sp. nov. is proposed.

### Description of *Streptomyces shinuiensis* sp. nov

*Streptomyces shinuiensis* (shin.ui.en’sis. N.L. masc. adj. *shinuiensis*, pertaining to Shinui, the island in the Republic of Korea, where the type strain was isolated).

Aerobic, Gram-stain-positive, oxidase-positive actinomycete forming an extensively branched, straight to flexuous substrate mycelium with aerial hyphae differentiating into straight to curved spore chains bearing oval-shaped spores (approximately 1.0 × 1.5 μm) with warty surface ornamentation. Good growth is observed on ISP2, ISP4, and marine agar; moderate growth on ISP1, ISP5, and ISP7; and poor growth on ISP3, ISP6, Czapek’s agar, and Gause’s synthetic agar no. 1. No aerial mycelium is observed on ISP3, ISP5, ISP6, or ISP7. Diffusible pigments are not produced. Growth occurs on ISP2 at 25–40°C (optimum, 30°C), at pH 7.0–9.0 (optimum, pH 8.0), and in the presence of 0–9% (w/v) NaCl (optimum, 3%). Starch, casein, and Tween 20 are hydrolyzed, but not elastin, cellulose, chitin, Tween 80, or DNA. Hydrogen sulfide is not produced. In the API 20NE system, positive results are observed for nitrate reduction and gelatin hydrolysis, while negative results are obtained for indole production, glucose fermentation, arginine dihydrolase, urease, esculin hydrolysis, and β-galactosidase (PNPG). In the API ZYM test, positive enzymatic activity is observed for alkaline phosphatase, esterase (C4), esterase lipase (C8), lipase (C14), leucine arylamidase, valine arylamidase, cystine arylamidase, and naphthol-AS-BI-phosphohydrolase. In the Biolog GEN III MicroPlate test, D-cellobiose, sucrose, D-raffinose, α-D-glucose, L-rhamnose, D-sorbitol, gelatin, L-lactic acid, and α-hydroxybutyric acid are oxidized. Cell wall contains *_L,L_*-diaminopimelic acid and glycine. The predominant cellular fatty acids are iso-C_16:0_ and anteiso-C_15:0_. The major polar lipids are phosphatidylethanolamine, diphosphatidylglycerol, and phosphatidylinositol dimannoside, two unknown phospholipids, one unknown glycolipid, and two unidentified lipids. The predominant respiratory quinone is menaquinone-9 (MK-9). The type strain, HK10^T^ (= KACC 22137^T^ = NBRC 114904^T^), was isolated from saltern sediment on Shinui Island, Republic of Korea.

The draft genome sequence of the type strain is 6.69 Mbp in length, with a DNA G+C content of 72.7%. The GenBank accession numbers for the 16S rRNA gene sequence and the draft whole-genome sequence are PV018052 and JBIHML000000000, respectively.

## Supplemental Materials

Supplementary data for this paper are available on-line only at http://jmb.or.kr.



## Figures and Tables

**Table 1:** Genomic characteristics of strain HK10^T^ and phylogenetically related type strains of the genus *Streptomyces*.

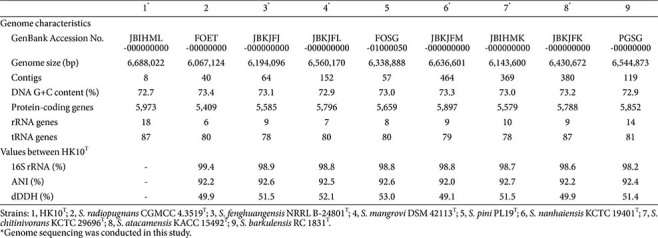

**Table 2:** Differential phenotypic characteristics of strain HK10^T^ and phylogenetically related type strains in the genus *Streptomyces*.

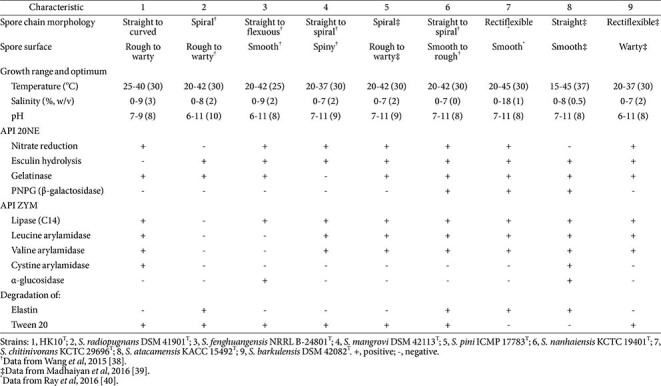

**Table 3:** Cellular fatty acid composition of strain HK10^T^ and related type strains of the genus *Streptomyces*.