# Supplementary material for: Streptomyces shinuiensis sp. nov., a Salternamides-Producing Bacterium Isolated from Saltern Sediment
Source: J Microbiol Biotechnol. 2025 Dec 18;35:e2510026. doi: 10.4014/jmb.2510.10026 (PMC12740849; doi:10.4014/jmb.2510.10026)
Supplement: Supplementary file 1 [file jmb-35-e2510026-supple.pdf]

J. Microbiol. Biotechnol. 2025. 35: e2510026

https://doi.org/10.4014/jmb.2510.10026

Supplementary Tables and Figure

Table S1. Genome assembly statistics of strain HK10^T and type strains of *Streptomyces* sequenced in this study.

	1	2	3	4	5	6
Total length	6,688,022	6,187,299	6,545,521	6,615,965	6,123,811	6,410,291
Number of contigs	8	64	152	464	369	380
Largest contig	5,931,859	675,741	441,237	160,764	161,598	239,365
N50	5,931,859	419,317	184,774	31,798	39,246	42,398
N90	198,609	190,158	54,892	9,120	11,716	11,348
L50	1	6	11	59	48	46
L90	2	15	35	200	154	158

Strains: 1, HK10^T; 2, *S. fenghuangensis* NRRL B-24801^T; 3, *S. mangrovi* DSM 42113^T; 4, *S. nanhaiensis* KCTC 19401^T; 5, *S. chitinivorans* KCTC 29696^T; 6, *S. atacamensis* KACC 15492^T.

Table S2. Biosynthetic gene clusters (BGCs) identified in the genome of strain HK10^T using antiSMASH v7.1.0. Each region is listed with its BGC type and the corresponding MiBIG reference (metabolite name, accession ID, and ClusterBlast similarity, when available). Note that a single BGC region may contain multiple biosynthetic types. ND, Not determined; no matching metabolite entry in the MIBiG database under the antiSMASH v7 settings. BGCs exhibiting >50% similarity to known clusters are shown in bold.

No.	Region	Type	MiBIG reference* (accession)	Similarity (%)
1	1.1	NRPS, NRPS-like	bonnevillamide D/ bonnevillamide E (BGC0002373)	10
2	1.2	terpene	ND	ND
3	1.3	NRPS, lanthipeptide-class-I, lanthipeptide-class-ii	ND	ND
**4**	**1.4**	**T2PKS, terpene, NRPS-like**	**spore pigment (BGC0000271)**	**66**
5	1.5	hglE-KS	Nataxazole (BGC0001213)	7
6	1.6	transAT-PKS-like, NRPS-like, T1PKS	cinnabaramide (BGC0000971)	18
**7**	**1.7**	**RiPP-like**	**Streptamidine (BGC0002115)**	**75**
8	1.8	terpene	Hopene (BGC0000663)	30
9	1.9	RiPP-like	ND	ND
**10**	**1.10**	**NRPS-like, PKS-like, T1PKS**	**sporolide A /sporolide B (BGC0000150)**	**68**
11	1.11	Ni-siderophore	Kanamycin (BGC0000236)	16
12	1.12	lanthipeptide-class-ii	ND	ND
13	1.13	LAP	Granaticin (BGC0000227)	10
14	1.14	Ni-siderophore	nonactin/ monactin/ dinactin /trinactin/ tetranactin (BGC0000244)	33
15	1.15	NRPS, T1PKS	Melanin (BGC0000909)	40
**16**	**1.16**	**lipolanthine, lanthipeptide-class-iii,**	**SapB (BGC0000551)**	**100**
**17**	**1.17**	**ectoine**	**Ectoine (BGC0002052)**	**100**
18	1.18	NRPS-like, RiPP-like	legonindolizidine A6 (BGC0002666)	20
19	1.19	terpene, NRPS, T1PKS	skyllamycin D/ skyllamycin E (BGC0002676)	21
**20**	**1.20**	**NI-siderophore, T3PKS**	**legonoxamine A/ desferrioxamine B /legono xamine B (BGC0002305)**	**100**
21	1.21	butyrolactone	ND	ND
22	1.22	NRPS, T1PKS	collismycin A (BGC0002305)	11
23†	4.1	HR-T2PKS, furan, nucleoside, butyrolactone	Asukamycin (BGC0000187)	14
24	5.1	NRPS, T1PKS	matlystatin A (BGC0001443)	11
25	6.1	T2PKS	lomaiviticin A/ lomaiviticin C/lomaiviticin D/ lomaiviticin E (BGC0000241)	36
26	6.2	butyrolactone	griseoviridin/ fijimycin A (BGC0000459)	8

* MIBiG stands for Minimum Information about a Biosynthetic Gene cluster. It is a curated database of experimentally characterized biosynthetic gene clusters with standardized annotations, including linked metabolite names and accession IDs.

† No. 23 (BGC region 4.1) is putatively responsible for salternamide biosynthesis

Table S3. Functional categorization of predicted protein-coding genes based on COG classification in the genome of strain HK10^T and *Streptomyces radiopugnans* CGMCC 4.3519^T. Code letters correspond to COG functional categories. Values represent the percentage of total protein-coding genes assigned to each category**.**

Strains: 1, HK10^T; 2, *S. radiopugnans* CGMCC 4.3519^T.

Code	Description	1	2
J	Translation, ribosomal structure, and biogenesis	6.6	6.7
K	Transcription	9.1	9.5
L	Replication, recombination, and repair	3.8	3.1
D	Cell cycle control, cell division, chromosome partitioning	2.1	1.9
V	Defense mechanisms	3.0	2.7
T	Signal transduction mechanisms	7.5	7.5
M	Cell wall/membrane/envelope biogenesis	4.7	5.3
U	Intracellular trafficking, secretion, and vesicular transport	1.0	0.9
O	Posttranslational modification, protein turnover, chaperones	3.9	4.2
C	Energy production and conversion	6.1	6.1
G	Carbohydrate transport and metabolism	7.8	8.5
E	Amino acid transport and metabolism	7.4	7.8
F	Nucleotide transport and metabolism	2.6	2.4
H	Coenzyme transport and metabolism	6.5	6.3
I	Lipid transport and metabolism	6.2	6.0
P	Inorganic ion transport and metabolism	4.4	4.8
Q	Secondary metabolites biosynthesis, transport, and catabolism	4.2	3.7
R	General function prediction only	8.3	8.3
S	Function unknown	5.3	2.8

Table S4. Carbon source oxidation pattern of strain HK10^T and related type strains of the genus *Streptomyces*.

Strains:1, HK10^T; 2, *S. radiopugnans* DSM 41901^T; 3, *S. fenghuangensis* NRRL B-24801^T; 4, *S. mangrovi* DSM 42113^T; 5, *S. pini* ICMP 17783^T; 6, *S. nanhaiensis* KCTC 19401^T; 7, *S. chitinivorans* KCTC 29696^T; 8, *S. atacamensis* KACC 15492^T; 9, *S. barkulensis* DSM 42082^T.

All strains were negative for D-turanose, stachyose, α-D-lactose, *N*-acetyl-*β*-D-mannosamine, *N*-acetyl-D-galactosamine, 3-methyl glucose, L-fucose, myo-inositol, D-fructose-6-PO_4, D-aspartic acid, D-serine, L-pyroglutamic acid, L-galactonic acid lactone, glucuronamide, quinic acid, p-hydroxy-phenylacetic acid, methyl pyruvate, D-lactic acid methyl ester, citric acid, α-keto-glutaric acid, D-malic acid, L-malic acid, bromo-succinic acid and formic acid. All data were obtained in this study. +, positive; –, negative; w, weakly positive.

Substrates	1	2	3	4	5	6	7	8	9
Dextrin	–	+	+	–	+	+	–	+	+
D-Maltose	–	+	+	–	–	+	+	–	+
D-Trehalose	–	+	–	–	–	+	+	+	–
D-Cellobiose	+	+	+	–	+	+	+	+	+
Gentiobiose	–	+	–	–	–	+	+	+	+
Sucrose	+	–	–	–	–	+	–	–	–
D-Raffinose	+	w	w	–	–	–	–	–	+
D-Melibiose	–	–	–	–	–	–	–	–	+
β-Methyl-D-Glucoside	w	–	w	–	–	–	–	–	+
N-Acetyl-D-Glucosamine	–	+	–	–	–	–	+	–	–
N-Acetyl-Neuraminic Acid	–	+	–	–	+	–	–	–	+
α-D-Glucose	+	+	+	–	–	+	–	+	+
D-Mannose	–	+	+	–	–	–	–	–	+
D-Fructose	–	–	+	–	–	–	–	–	+
D-Galactose	–	–	w	–	–	–	–	–	+
D-Fucose	–	–	–	–	–	–	–	–	+
L-Rhamnose	+	+	+	–	–	–	–	–	+
Inosine	–	–	–	–	w	–	–	+	–
D-Sorbitol	+	–	w	–	–	–	–	–	–
D-Mannitol	w	–	–	–	–	–	+	–	–
D-Arabitol	w	+	w	–	w	–	+	–	+
Glycerol	–	+	+	–	–	+	+	+	+
D-Glucose-6-PO_4	–	–	–	–	–	–	+	–	+
Gelatin	+	–	+	–	w	+	–	+	+
Glycyl-L-Proline	–	+	+	–	–	–	–	+	+

L-Alanine	–	+	+	+	+	–	–	+	–
L-Arginine	–	–	–	–	–	+	–	–	w
L-Aspartic Acid	–	+	+	–	–	+	–	+	+
L-Glutamic Acid	–	+	+	–	–	–	+	+	–
L-Histidine	–	+	+	–	+	+	–	+	–
L-Serine	–	+	+	–	+	–	–	+	+
D-Galacturonic Acid	–	–	+	–	–	–	–	–	w
D-Gluconic Acid	–	+	–	–	–	+	+	–	+
D-Glucuronic Acid	–	–	w	–	–	–	–	–	+
Mucic Acid	–	–	–	–	–	+	–	–	w
D-Saccharic Acid	–	+	w	–	–	–	–	+	w
L-Lactic Acid	+	–	–	–	–	–	–	–	–
Tween 40	–	+	+	–	+	+	+	+	+
γ-Amino-Butryric Acid	–	+	+	–	w	+	+	+	–
α-Hydroxy-Butryric Acid	+	–	-	–	–	–	–	–	–
β-Hydroxy-D, L-Butryric Acid	–	+	+	–	–	–	+	+	+
α-Keto-Butryric Acid	–	–	+	–	w	–	–	–	–
Acetoacetic Acid	–	+	+	–	w	–	+	–	–
Propionic Acid	–	+	+	–	–	+	+	+	+
Acetic Acid	–	+	+	–	–	+	+	+	+

Table S5. Cultural characteristics of strain HK10^T grown on various agar media, including standard ISP formulations recommended for *Streptomyces* classification.

Growth intensity and pigmentation of aerial and substrate mycelia were evaluated after incubation at 30 °C for 20 days.

		Color of:	
**Agar medium**	**Growth**	**Aerial mycelium**	**Substrate mycelium**
ISP1 (Trypton/yeast extract)	Moderate	White	Pale yellow
ISP2 (Yeast extract/malt extract)	Good	Grey	Yellow
ISP3 (Oatmeal)	Poor	None	None
ISP4 (Inorganic salts/starch)	Good	Grey	Yellow
ISP5 (Glycerol/asparagine)	Moderate	None	Pale yellow
ISP6 (Peptone/yeast extract/iron)	Poor	None	None
ISP7 (Tyrosine)	Moderate	None	Yellow
Marine agar 2216	Good	Grey	Pale yellow
Czapek's agar	Poor	White	Yellow
Gause's synthetic agar no.1	Poor	White	Yellow

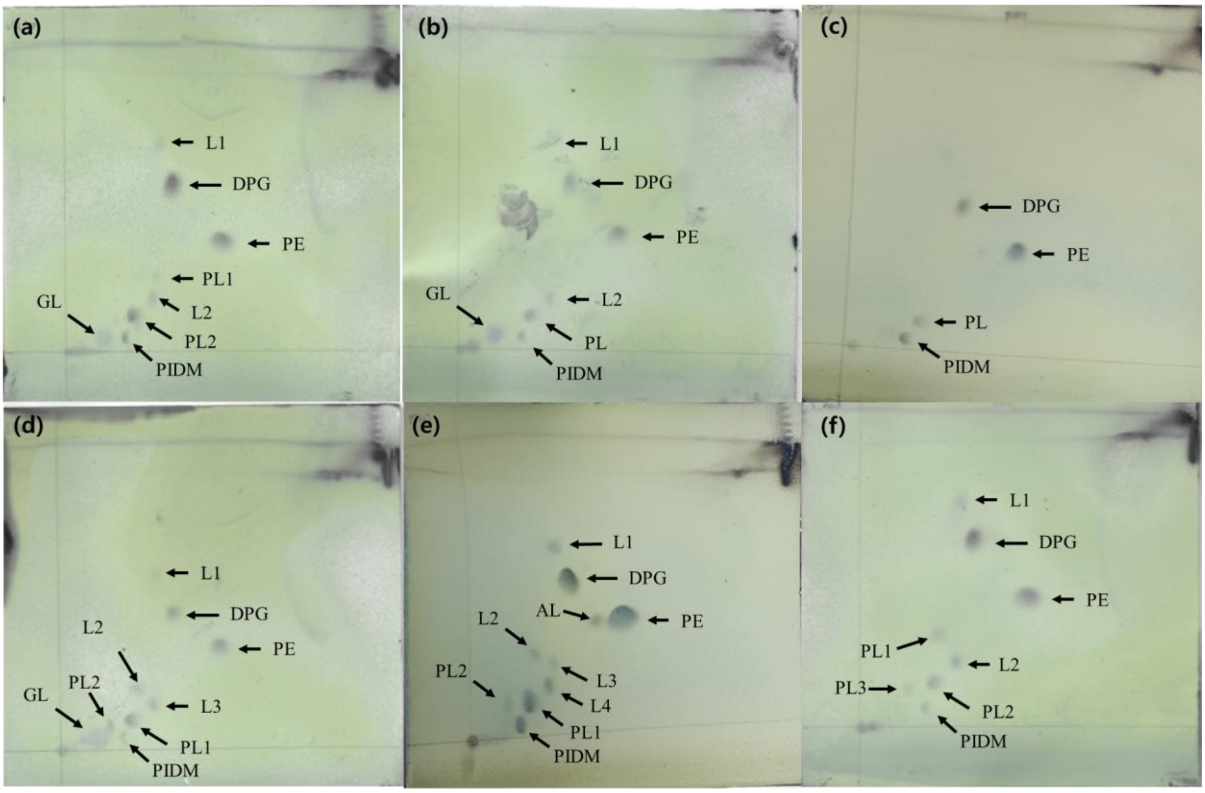

Fig. S1. Two-dimensional thin-layer chromatograms of total polar lipids of strain HK10^T (a), *S. radiopugnans* DSM 41901^T (b), *S. fenghuangensis* NRRL B-24801^T (c), *S. mangrovi* DSM 42113^T (d), *S. pini* ICMP 17783^T (e), and *S. barkulensis* DSM 42082^T (f). Abbreviations: PE, phosphatidylethanolamine; DPG, diphosphatidylglycerol; PIDM, phosphatidylinositol dimannoside; PL, unknown phospholipid; GL, unknown glycolipid; L1-L4, unidentified lipids.
